# Assessing amino acid racemization variability in coral intra-crystalline protein for geochronological applications

**DOI:** 10.1016/j.gca.2012.02.020

**Published:** 2012-06-01

**Authors:** Erica J. Hendy, Peter J. Tomiak, Matthew J. Collins, John Hellstrom, Alexander W. Tudhope, Janice M. Lough, Kirsty E.H. Penkman

**Affiliations:** a School of Earth Sciences, University of Bristol, Bristol, BS8 1RJ, United Kingdom; b School of Biological Sciences, University of Bristol, Bristol, BS8 1UG, United Kingdom; c BioArCh, Departments of Archaeology and Chemistry, University of York, York, YO10 5DD, United Kingdom; d School of Earth Sciences, University of Melbourne, Melbourne, VIC 3010, Australia; e School of GeoSciences, University of Edinburgh, Edinburgh EH9 3JW, UK; f Australian Institute of Marine Science, PMB3, Townsville M.C., QLD 4810, Australia

## Abstract

Over 500 Free Amino Acid (FAA) and corresponding Total Hydrolysed Amino Acid (THAA) analyses were completed from eight independently-dated, multi-century coral cores of massive *Porites* sp. colonies. This dataset allows us to re-evaluate the application of amino acid racemization (AAR) for dating late Holocene coral material, 20 years after Goodfriend et al. (*GCA***56** (1992), 3847) first showed AAR had promise for developing chronologies in coral cores. This re-assessment incorporates recent method improvements, including measurement by RP-HPLC, new quality control approaches (e.g. sampling and sub-sampling protocols, statistically-based data screening criteria), and cleaning steps to isolate the intra-crystalline skeletal protein. We show that the removal of the extra-crystalline contaminants and matrix protein is the most critical step for reproducible results and recommend a protocol of bleaching samples in NaOCl for 48 h to maximise removal of open system proteins while minimising the induced racemization. We demonstrate that AAR follows closed system behaviour in the intra-crystalline fraction of the coral skeletal proteins. Our study is the first to assess the natural variability in intra-crystalline AAR between colonies, and we use coral cores taken from the Great Barrier Reef, Australia, and Jarvis Island in the equatorial Pacific to explore variability associated with different environmental conditions and thermal histories. Chronologies were developed from THAA Asx D/L, Ala D/L, Glx D/L and FAA Asx D/L for each core and least squares Monte Carlo modelling applied in order to quantify uncertainty of AAR age determinations and assess the level of dating resolution possible over the last 5 centuries. AAR within colonies follow consistent stratigraphic aging. However, there are systematic differences in rates between the colonies, which would preclude direct comparison from one colony to another for accurate age estimation. When AAR age models are developed from a combined dataset to include this natural inter-colony variability THAA Asx D/L, Glx D/L and Ala D/L give a 2σ age uncertainty of ±19, ±38 and ±29 year, for the 20th C respectively; in comparison 2σ age uncertainties from a single colony are ±12, ±12 and ±14 year. This is the first demonstration of FAA D/L for dating coral and following strict protocols 2σ precisions of ±24 years can be achieved across different colonies in samples from the last 150 years, and can be ±10 years within a core from a single colony. Despite these relatively large error estimates, AAR would be a valuable tool in situations where a large number of samples need to be screened rapidly and cheaply (e.g. identifying material from mixed populations in beach or uplift deposits), prior to and complementing the more time-consuming geochronological tools of U/Th or seasonal isotopic timeseries.

## 1 Introduction

Climate reconstructions from corals are an important tool for documenting tropical climate on timescales from individual river flood events to centennial and millennial variability (e.g. Tudhope et al., 2001; Hendy et al., 2002; Cobb et al., 2003; Abram et al., 2008, 2009; Lough, 2011). The aragonitic skeletons of massive *Porites* colonies can archive continuous high-resolution proxy records over a multi-century lifespan. Understanding the spatial patterns and mechanisms of climate variability requires absolute age control of these records. Current approaches to dating recent coral-climate records collected from living colonies (i.e. within the last 500 years) include counting annual markers. For example, skeletal density banding visible in X-radiographs (first demonstrated by Knutson et al., 1972), luminescence banding visible under UV light with the application of cross-dating techniques adapted from dendrochronology (Hendy et al., 2003), and seasonal cycles in high resolution sampled trace element and δ^18^O records (e.g. Tudhope et al., 1995). However, in locations with low-amplitude seasonality in temperature and/or rainfall these band-counting techniques are considerably more challenging. In addition, recently dead coral colonies may provide important palaeoenvironmental records providing their age is known. The best radiometric dating technique on this timescale is U/Th (Gale, 2009). However, providing a U-series chronology for recent coral records is a particular challenge (e.g. Cobb et al., 2003; Yu et al., 2006) due to the limited radioactive decay of ^234^U to ^230^Th over such a short time frame and the proportionally larger correction required for site-specific non-radiogenic ^230^Th (Shen et al., 2008; Zhao et al., 2009).

In this paper we evaluate the application of amino acid racemization (AAR) of intra-crystalline protein as an alternative supplementary technique for dating recent coral records. AAR is the slow inter-conversion (racemization) of l-amino acids, the basic building blocks of protein, into an equilibrium mixture of l- and d-amino acids with time (reviewed in Goodfriend et al., 2000). Consistent racemization trends have been observed in two coral-based AAR studies, each performed on the whole protein fraction of a single living scleractinian coral colony (Goodfriend et al., 1992; Nyberg et al., 2001). However, Husseini (1973) and Wehmiller et al. (1976) found significant variations in racemization data between multiple older (Pleistocene) corals which resulted in non-concordant ages, with leaching, recrystallisation and/or contamination suggested as the cause. It has been argued that isolating the intra-crystalline fraction of protein from biominerals would provide a more coherent fraction for the study of protein breakdown (i.e. a closed system with predictable kinetics unaffected by external factors in the burial environment) (Towe, 1980; Sykes et al., 1995; Collins and Riley, 2000; Ingalls et al., 2003; Penkman et al., 2008). A new technique of amino acid analysis has been developed for geochronological purposes (Penkman, 2005; Penkman et al., 2008) that combines reverse-phase high-pressure liquid chromatography (RP-HPLC) analysis (Kaufman and Manley, 1998) with the isolation of the ‘intra-crystalline’ fraction of amino acids by bleach treatment (Sykes et al., 1995). This approach, termed intra-crystalline protein decomposition (IcPD), provides D/L values of multiple amino acids from the chemically-protected protein within the biomineral, reduces the amount of sample needed and increases analytical reliability. The method is also cost-effective for analysis of a large number of specimens.

Here we investigate the potential of intra-crystalline AAR as a dating technique in eight independently-dated, multi-century coral cores of massive *Porites* sp. By focusing on the intra-crystalline fraction (operationally defined as the organic matter that is resistant to strong chemical oxidation, e.g. Towe and Thompson, 1972), we avoid including any ‘open system’ material or external sources of contamination in our analyses. Our study is the first to assess the natural variability in intra-crystalline AAR between colonies and hence the level of dating resolution possible in coral for the last 5 centuries. AAR was also examined in both the Free Amino Acid (FAA) and corresponding Total Hydrolysable Amino Acid (THAA) fractions. Using this large dataset, we are also able to review methods of assessing AAR data quality and to further develop screening criteria for excluding outlier measurements or samples (Kosnik and Kaufman, 2008; Kosnik et al., 2008). In addition, we assess the utility of a least squares Monte Carlo approach that uses the internal stratigraphy of the coral to constrain the age estimates from AAR.

## 2 Materials and methods

### 2.1 Coral core samples

**Fig. 1:**
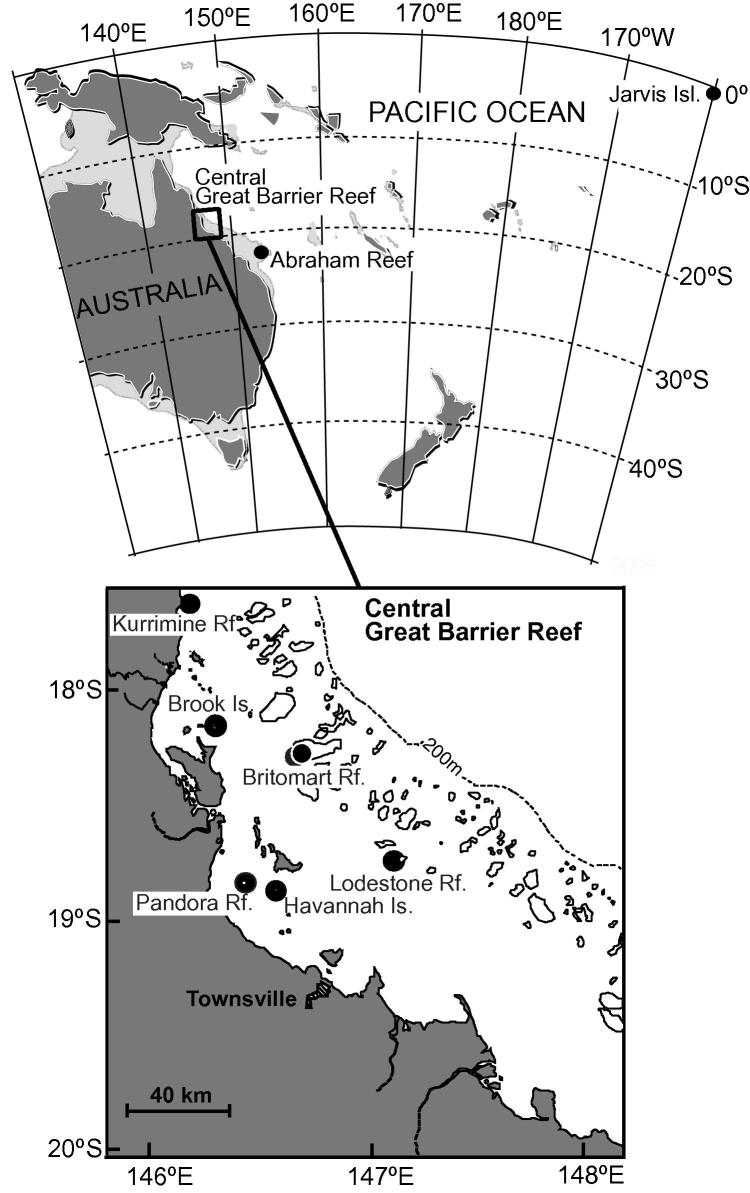
Massive *Porites* coral core sample sites; 8 from this study and Abraham Reef (Goodfriend et al., 1992).

Seven multi-century long cores were taken from massive *Porites* coral colonies at six inshore and mid-shelf reefs in the central Great Barrier Reef (GBR), Australia, and one core from a colony at Jarvis Island in the central equatorial Pacific (Table 1; Fig. 1). The cores span 134–422 years of colony growth. The GBR cores were independently dated in a previous study (Hendy et al., 2003) using a combination of annual density banding from X-radiographs of the 7 mm thick coral slices and cross-dating using characteristic patterns of luminescent lines seen when the corals were placed under UV light. The luminescent lines are associated with the Australian summer monsoon rains and thereby provide a strong seasonal marker with characteristic patterns between years (Isdale, 1984). Typical errors found for coral core age estimates from annual banding are 1–2 years/century (Hendy et al., 2003). For the seven GBR coral cores a ‘master’ chronology was developed using cross-dating techniques to strengthen the dating control between individual cores (Hendy, 2003). For the Jarvis coral, a chronology was established using a combination of seasonal and ENSO-related variations in monthly-resolved skeletal δ^18^O records, and annual skeletal density banding. Any differences in AAR between coral cores are therefore unlikely to be caused by incorrect assignment of sample age.

Core slices were subjected to repeated and focused ultrasonic cleaning in high-purity 18 Ω cm water (Milli-Q) water and dried at <40 °C for a maximum of 48 h. Skeletal material was milled from along the centre of a growth axis to produce a fine powder. Duplicates for the same time period were collected from adjacent growth axes. The majority of samples contain a 5-year increment of skeletal growth, but a series of high resolution (sub-annual) analyses were taken at the base, middle and coral top of the Jarvis core. Since five species of massive *Porites* are known to occur within the regions covered in this study (*P. lutea* and *P. lobata* are the most common, but *P. australiensis, P. mayeri* and P. *solida* are also recorded), a number of species were potentially collected within this sample set. Massive *Porites* sp. are notoriously difficult to identify accurately to species level, especially in archived core material, and so we do not have access to this level of detail. It should be noted, however, that skeletal properties have been examined between massive *Porites* species and results rarely support any species-based differences (e.g. Lough and Barnes 1992, 2000), and so it is usual practice to treat massive *Porites* sp. as one population (e.g. Lough and Barnes, 2000).

### 2.2 Isolation of intra-crystalline fraction

**Fig. 2:**
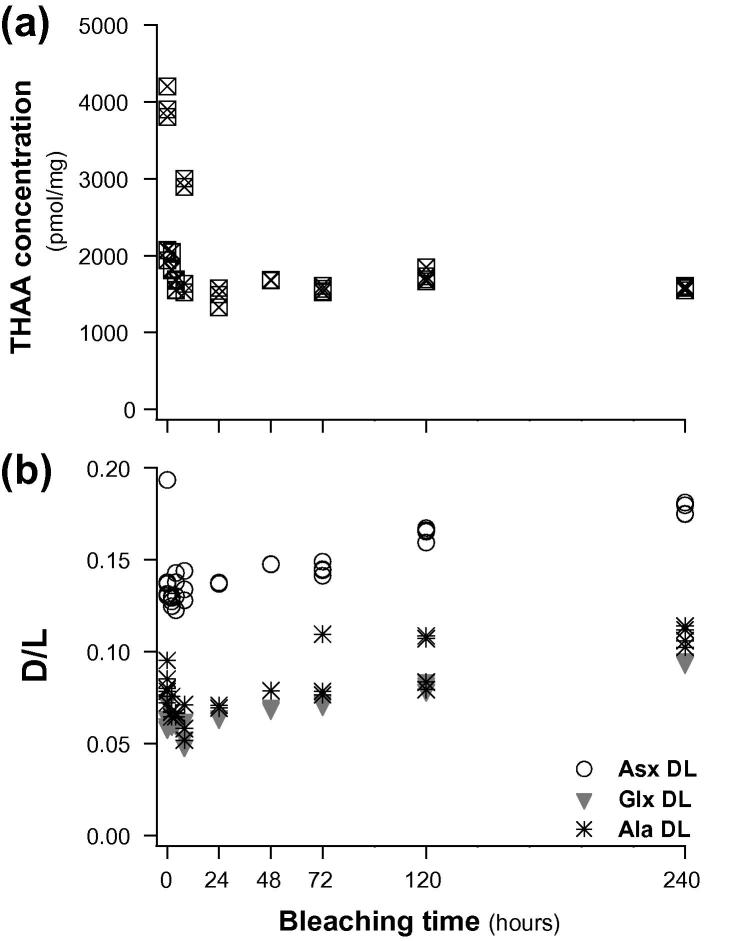
(a) Decline in the total hydrolysable amino acid (THAA) concentration in modern *Porites* with oxidation time using NaOCl. The intra-crystalline fraction is the residual fraction of amino acids that are not oxidised. (b) Change in Asx, Glx and Ala D/L in the THAA with oxidation time; a slight increase is observed after prolonged bleaching.

Protein degradation must occur within a ‘closed system’ for AAR to provide geochronological information (see discussion in Collins and Riley, 2000). Ingalls et al. (2003) found good preservation of the intra-crystalline organic matter in corals after treatment with 5% NaOCl and postulated that this could be a reliable source of ‘closed system’ material for coral-based studies. Since the variability identified in earlier coral AAR studies was proposed to have been caused by contamination and leaching, we undertook a preliminary study to establish a protocol for isolating the intra-crystalline fraction. A stable intra-crystalline fraction of protein has been isolated in mollusc shells and eggshells by oxidation with 12% NaOCl (Penkman et al., 2008; Demarchi et al., in press). We therefore determined the optimum treatment time for *Porites* coral by oxidising exposed organics in powdered skeletal samples (<100 μm) with 12% NaOCl (50 μL/mg of sample) for varying times up to 10 days. After ∼24 h of bleaching AA concentrations decreased to a relatively stable plateau (Fig. 2a). However, an increase in the extent of racemization was also observed as bleaching time increased (Fig. 2b), possibly due to sequential etching of the biomineral by the NaOCl. Therefore, the optimum sample protocol selected (and applied in all further analyses) was to bleach samples in 12% NaOCl (50 μL/mg of sample) for 48 h, maximising removal of the inter-crystalline proteins but minimising induced racemization.

### 2.3 Amino acid analysis

Proteins must be broken down into their constituent amino acids prior to analysis by RP-HPLC. This occurs spontaneously over time by a process of peptide bond hydrolysis and, as a result, it is possible to analyse the naturally hydrolysed (free) amino acids in degraded samples, although residual peptides will remain undetected. Concentrated mineral acid at high temperature is typically used to hydrolyse all residual peptide bonds within a sample. Analysis of intra-crystalline samples both with and without this complete hydrolysis step gives two alternative composition measures of the amino acid stereoisomers: the Free Amino Acid (FAA) fraction and the Total Hydrolysable Amino Acid (THAA) fraction. Undertaking both analyses reveals further aspects of protein degradation and potentially useful indicators of age, for example, the extent of peptide bond hydrolysis and decomposition of the free amino acids, in addition to the extent of racemization.

All samples were prepared following the optimised bleaching protocol established above (Section 2.2) and the methods of Penkman et al. (2008). In brief, each sample (∼3 mg) was powdered and bleached for 48 h with 12% NaOCl. Two subsamples were taken: one fraction was directly demineralised and the free amino acids (FAA) analysed, and the second was treated with 7 M HCl under N_2_ at 110 °C for 24 h (H*) to release the peptide-bound amino acids, thus yielding the ‘total hydrolysable’ amino acid concentration (THAA). Samples were then dried by centrifugal evaporator and rehydrated for RP-HPLC analysis with 0.01 mM l-homo-arginine as an internal standard.

The amino acid compositions of the samples were analysed in duplicate by RP-HPLC using fluorescence detection following a modified method of Kaufman and Manley (1998). Two μl of sample was injected and mixed online with 2.2 μl of deriviatising reagent (260 mM *n*-Iso-l-butyryl l-cysteine (IBLC), 170 mM o-phthaldialdehyde (OPA) in 1 M potassium borate buffer, adjusted to pH 10.4 with KOH pellets). The amino acids were separated on a C18 HyperSil BDS column (5 mm × 250 mm) at 25 °C using a gradient elution of three solvents: sodium acetate buffer (solvent A; 23 mM sodium acetate tri-hydrate, 1.5 mM sodium azide, 1.3 μM EDTA, adjusted to pH 6.00 ± 0.01 with 10% acetic acid and sodium hydroxide), methanol (solvent C) and acetonitrile (solvent D). The l and d isomers of 10 amino acids were analysed routinely. It is not possible to distinguish between the acidic amino acids and their amine derivatives because both asparagine and glutamine undergo rapid irreversible deamination during preparative hydrolysis to aspartic acid and glutamic acid, respectively (Hill, 1965). Aspartic acid and asparagine are therefore reported together as Asx, and glutamic acid and glutamine as Glx.

### 2.4 Amino acid data screening

The relative stability of different amino acids varies, but both concentration and extent of racemization of two different amino acids should covary in a systematic manner. Any deviation from covariance provides a means of identifying compromised samples (e.g. Kaufman, 2003, 2006; Laabs and Kaufman, 2003; Kosnik and Kaufman, 2008). D/L values of the FAA and THAA fractions for individual amino acids should also covary in a consistent manner if racemization is occurring within a closed system. Deviation in these cases indicate samples have been compromised, for example, by bacterial contamination or recrystallisation (e.g. Miller and Brigham-Grette, 1989; Preece and Penkman, 2005).

**Fig. 3:**
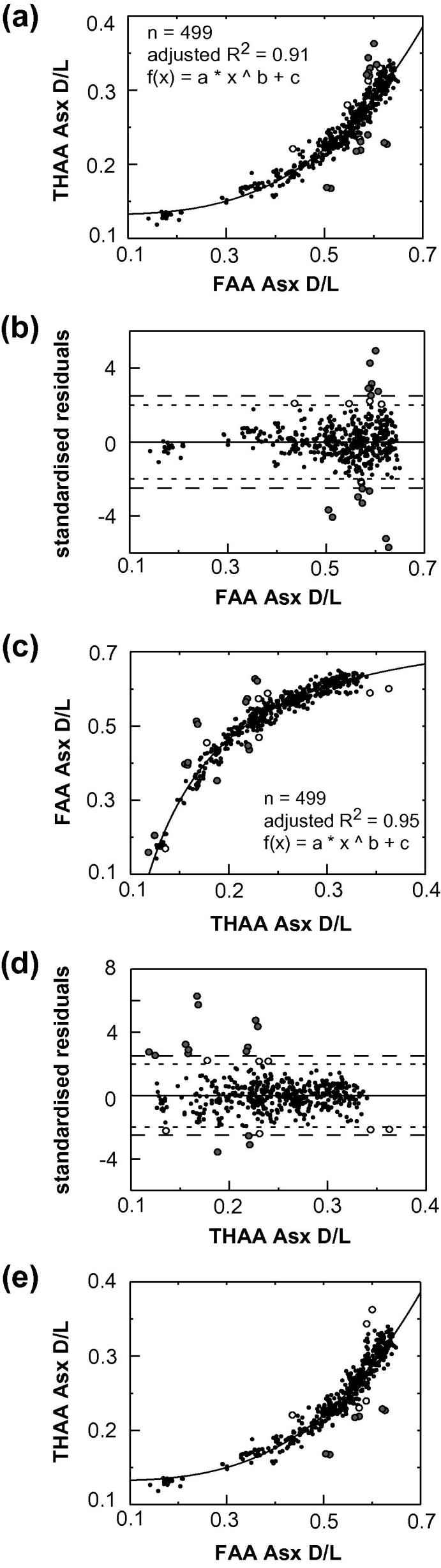
(a) and (c) The relationship between FAA and THAA Asx D/L, with fitted power function. Insert gives adjusted R^2^ value, sample size (*n*) and fitted function. (b) and (d) display the standardised residuals calculated from the relationship depicted in (a) and (c), respectively. The larger and smaller dashed lines delimit the 2.5 stdev and 2.0 stdev cut-off values, respectively. (e) The relationship between Free and Hydrolysable Asx D/L identifying only outliers highlighted in both regression analyses independent of which variable was assigned to the *x*-axis. In all graphs, grey circles identify outliers using a 2.5 stdev cut-off point, white circles are additional data-points designated as outliers using 2.0 stdev.

**Fig. 4:**
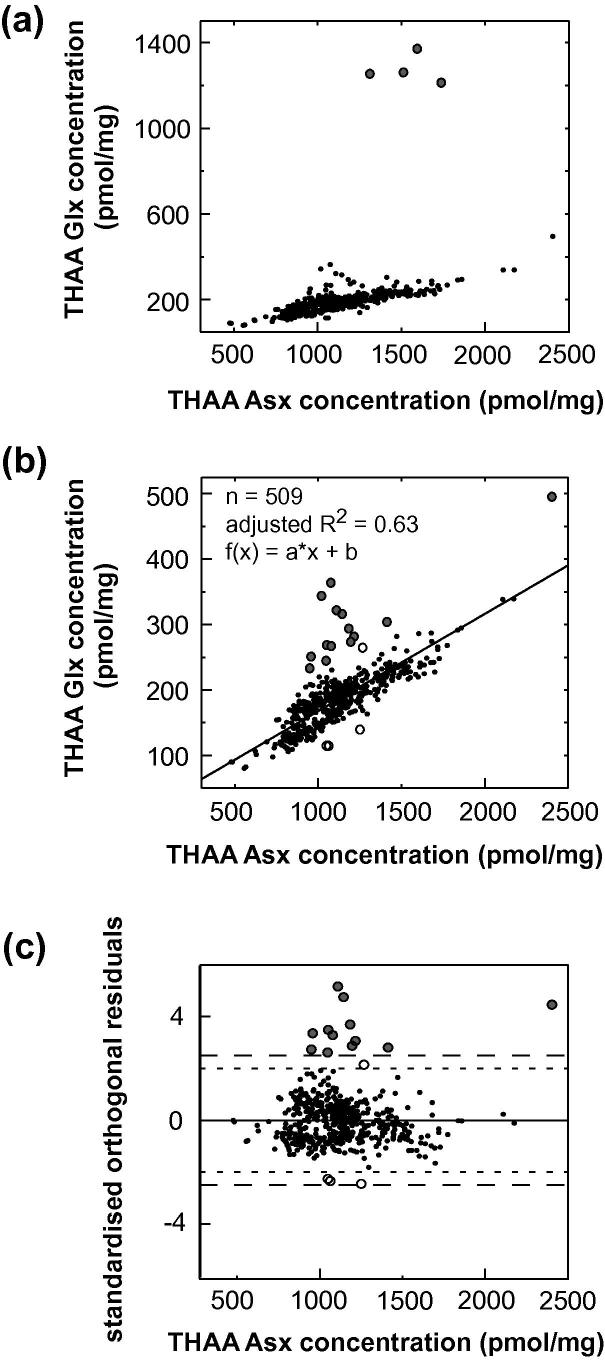
(a) THAA Asx vs Glx concentration. Four-data points (grey circles) were excluded from the data-set prior to statistical analysis. (b) Asx vs Glx concentration, with orthogonal distance regression fitted-line and outliers highlighted. Insert gives adjusted R^2^ value, sample size (*n*) and fitted function. (c) Standardised residuals calculated from the relationship depicted in (b), with outliers highlighted. The larger and smaller dashed lines delimit the 2.5 stdev and 2.0 stdev cut-off values, respectively. In graphs (b) and (c), data-points with standardised residuals >2.5 stdev are represented by grey circles, while data-points with standardised residuals between 2 and 2.5 stdev, and so only designated as outliers when the more stringent cut-off value of 2.0 stdev is applied, are represented by white circles.

**Fig. 5:**
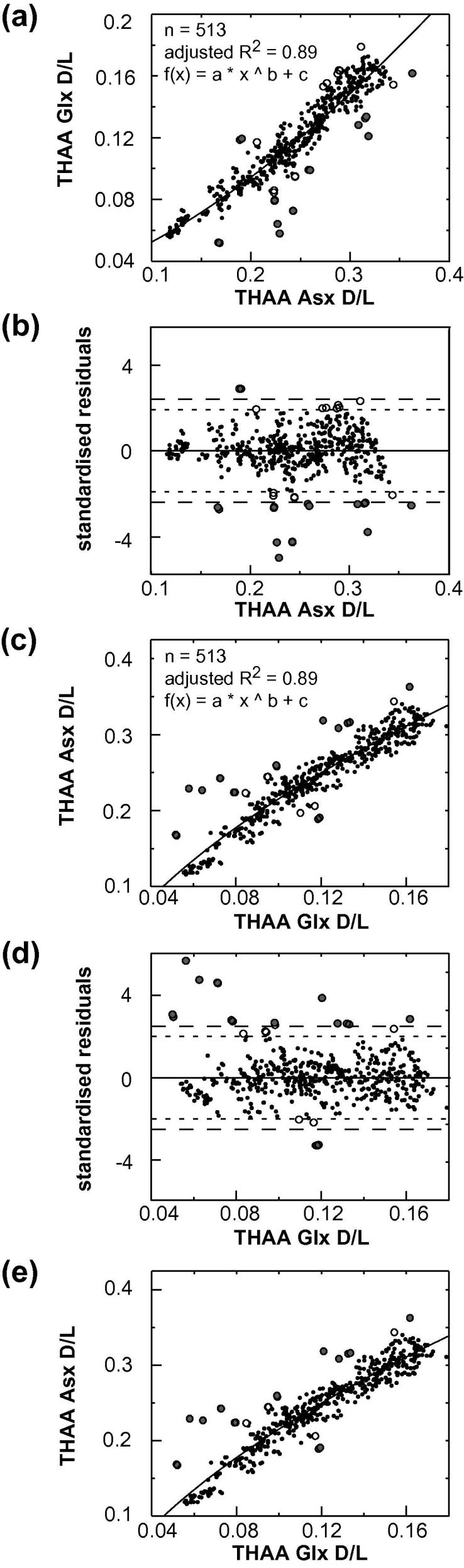
(a) and (c) THAA Asx D/L vs THAA Glx D/L, with the fitted power function. Insert gives adjusted R^2^ value, sample size (*n*) and fitted function. (b) and (d) display the standardised residuals calculated from the relationship depicted in (a) and (c), respectively. (e) The relationship between THAA Asx D/L and THAA Glx D/L with data-points only designated as outliers if highlighted by the statistical tests irrespective of the variable placed on each axis. In all graphs, data-points with standardised residuals >2.5 stdev are represented by grey circles, while data-points with standardised residuals between 2 and 2.5 stdev, and so only designated as outliers by the 2.0 stdev cut-off value, are represented by white circles.

A series of data quality tests were applied to exclude data points that clearly indicate compromised samples, while retaining the natural variability in the dataset. Covariance was examined between (i) the D/L values of the FAA and the THAA fraction of Asx (Fig. 3), (ii) the concentration of Asx and Glx, (Fig. 4) and (iii) the D/L values of Asx and Glx (Fig. 5). Outliers were identified following the guidelines devised by Kosnik and Kaufman (2008); regression analysis was performed (using Matlab version 7.10.0), and data-points with residuals above or below a specified cut-off point were regarded as outliers. However, because simple regression analysis involves minimising the sum of the squared vertical distances between the fitted line and *y*-values, this test assumes that error is exclusive to the variable plotted on the *y*-axis (i.e. ‘dependent variable’). All variables used in the data-screening tests have some associated uncertainty and, as a result, different data-points can be identified as outliers, depending on which of the two variables under examination is (arbitrarily) assigned to either the *x* or *y*-axis. Therefore, we applied orthogonal-distance regression (ODR) in place of linear regression. ODR minimises the perpendicular distances (rather than the vertical) between *y*-values and the fitted line, thereby taking into account uncertainty associated with both variables. Due to the complexity of using ODR for non-linear functions, where the data were non-linear, regression analysis was performed twice, switching the variable assigned to the *y*-axis (i.e. dependent variable) between tests, with only those data-points designated as outliers on both occasions excluded from the data set.

Function selection for each regression analysis was based on a set of criteria including: optimal adjusted R^2^ values, comparisons using the F-test or Akaike’s information criterion (using GraphPad Prism, version 5.0), and whether the shape of the regression-line conformed with *a priori* knowledge regarding the reaction taking place. Data points with residuals outside the specified cut-off values (2.5 and 2 stdev) are highlighted in the data screening plots (Figs. 3–5). Close examination of the raw D/L and concentration data for these individual points demonstrated that a cut-off value of 2.5 stdev was most appropriate for identifying “anomalous” measurements (i.e. possessed THAA concentrations that were visibly outside of the remaining data-set). The data screening tests described in Sections 2.4.1–3, with the application of a 2.5 stdev cut-off value, resulted in the exclusion of 28 data points from the total AAR data set (in which analytical replicates were counted as individual data-points) of >500 (5%). Using the 2.0 stdev cut-off value, the number of data-points regarded as outliers increased to 35. In addition, evidence of poor replication between analytical duplicates, and extreme THAA concentrations, were also used to screen data (Section 2.4.4; Electronic Annex, EA). The exclusion rate was slightly lower than previous studies using different screening criteria (e.g. Kaufman, 2003, 2006).

#### 2.4.1 FAA Asx D/L vs THAA Asx D/L

Predicting the pattern of covariance between the D/L values in the free and total hydrolysable amino acid fractions is not simple because of the combination of different rate constants and activation energies for the AAR reactions. Although racemization of FAA appears to follow reverse first-order kinetics in aqueous solutions (Bada, 1971), this is not the case in fossil samples (e.g. Kriausakul and Mitterer, 1980) because as protein diagenesis proceeds, peptide bond hydrolysis introduces additional amino acids to the FAA pool, which may have already undergone racemization whilst in-chain or terminal. The THAA fraction represents an even more complex situation, as bound amino acids can racemize at either the N- or C- termini of the protein chain, or, if existing as dipeptides, can undergo rapid racemization via the formation of diketopiperazines (DKPs; Steinberg and Bada, 1981). In addition, some amino acids, such as aspartic acid, can racemize within-chain via the formation of cyclic succinimide molecules (Geiger and Clarke, 1987; Radkiewicz et al., 1996; Collins et al., 1999). Despite such complications, racemization should progress with D/L values increasing with time. Therefore, if a function produced a best-fit regression line possessing a negative gradient at any point, it was rejected. The power function gave the best “fit” to the FAA and THAA Asx D/L data (adjusted R^2^; Fig. 3a and c).

#### 2.4.2 THAA Asx concentration vs THAA Glx concentration

The concentration of THAA Asx and Glx declined linearly over the time period considered (Fig. 4a and b), as also found by Kosnik and Kaufman (2008) in late Holocene shells of four molluscan taxa. Four data-points deviated considerably (Fig. 4a) and were excluded prior to the statistical-tests because they severely compromised the application of the regression-based outlier-detection method.

#### 2.4.3 THAA Asx D/L vs THAA Glx D/L

These two variables follow complex racemization kinetics (Section 2.4.1). Both non-linear and linear functions were examined, with the non-linear power function providing the best fit (adjusted R^2^ values; Fig. 5a and c).

#### 2.4.4 Analytical replicates

FAA and THAA analyses were duplicated for all samples and measurements were run on different days (following standard protocol; Penkman, 2005). Analytical duplicates with differences greater than the 4 stdev of differences recorded for the whole population of >500 samples were investigated further. If the duplicates of only one amino acid were identified in this manner, then just the values for that amino acid of both duplicates were removed. If, however, duplicates of more than one amino acid measurement were different by >4 stdev, then all values were excluded from the data set. The exclusion of anomalous analytical duplicates resulted in the removal of 55 values, of which eight involved complete removal of all data for the analytical sample. An additional four samples were excluded as outliers due to their extreme high/low concentrations (>2 stdev from the average THAA for the full data set; see EA for details).

### 2.5 Treatment of data from previous study to enable direct comparison

Massive *Porites* coral skeleton has been studied for amino acid geochronology in one previous study (Goodfriend et al., 1992). This single core from Abraham Reef, a site ∼200 miles further south of our study area (Fig. 1), is likely to have experienced similar relative SST seasonality and seawater pH conditions to the seven other GBR cores, although slightly cooler SSTs on average (Table 1). In order to directly compare the data obtained from our samples with the Goodfriend et al. study, there are three factors which need to be examined: (a) the differences in chromatographic method used; (b) the time between collection and analysis and (c) the difference in hydrolysis methods.

#### 2.5.1 Chromatographic method differences

Asx was measured by gas chromatography in the Abraham core (Goodfriend et al., 1992) and RP-HPLC in this study. An inter-laboratory comparison to test the detection of d and l aspartic acid between GC and RP-HPLC analyses was undertaken with the Institut für Rechtsmedizin laboratory in Kiel (Penkman, 2005; GC data from R. Dobberstein & S. Ritz-Timme). The D/L Asx obtained ranged from 0.016 to 0.956, thus accounting for almost the full range of natural samples from living tissue to amino acids at equilibrium. A correlation coefficient of 0.9998 (*n* = 16) was obtained on cross-comparison of the data. The relationship was not exactly 1:1, instead the RP-HPLC method yielded slightly higher D/L Asx at values <0.34 relative to the GC method, but the difference was not significant and, therefore, no correction was applied.

#### 2.5.2 Time between collection and analysis

The Abraham coral was collected in December 1985, the Pandora core on 24th March 1984 and Havannah on 8th June 1988. The cores analysed in this study were kept in controlled temperature conditions at about 20 °C from 1997. Although it is not known exactly when the Goodfriend analyses were undertaken, it must have been before May 1992, so the maximum time that the samples were kept before analysis was 7 years. In contrast, the Pandora and Havannah cores were not analysed until 21 and 17 years after collection respectively. As a result, the minimum time between our analyses and those of Goodfriend was 13 years. The initial rates of Asx racemization are very rapid, so we have offset the Goodfriend dataset by 13 years to take account of the minimum level of protein breakdown that would have been observed in the Abraham coral sample if it had been analysed at the same time as the coral cores in this study.

#### 2.5.3 Hydrolysis method differences

For the amino acids to be detected using these analytical methods, they must be in the free form, achieved by the preparative hydrolysis step. The increase in the extent of hydrolysis induced racemization between this study (7 M HCl @ 110 °C for 24 h), and that of Goodfriend et al. (1992) (6 M HCl @ 150 °C for 15 min) was estimated to be D/L of 0.043 when D/L = 0. This was estimated assuming an activation energy of 83 kJ mol^−1^ derived from data supplied by Csapo et al. (1997). We made no correction for differences in molarity of HCl, which in our study is approximately 6 M after dissolution of the carbonate.

In the following figures the Goodfriend et al. (1992) data have been offset to include both the corrections made for the differences in hydrolysis induced racemization and in time between collection and analysis.

### 2.6 Least squares Monte Carlo modelling of AAR age estimates

An interpolated model of racemization with depth down core improves the practical geochronological use of AAR. We apply a least squares Monte Carlo approach previously derived for fitting age-depth models to an analogous system of U/Th data vs depth in speleothem records (e.g. Drysdale et al., 2004, 2005). The method fits a continuous sequence of line segments between adjacent measurements, subject to the constraint that amino acid D/L must always increase with time, using an uncertainty-weighted least squares technique. The model also seeks to minimise relative change in racemization rate between measurements, subject to measurement uncertainty, which has the effect of smoothing its D/L vs time output. The model is then run many thousands of times, randomising the input D/L data subject to their uncertainties at each iteration, allowing median, upper and lower 95% confidence interval curves can be calculated. Similarly to Drysdale et al. (2005), each iteration of this Monte-Carlo simulation also varies the racemization rate between adjacent measurements, such that interpolation uncertainty is accounted for where analyses are widely spaced. Uncertainties for individual analyses were obtained for each modelled data series by determining the standard deviation where multiple analyses were obtained for a single year of that series. This value was then assigned to all analyses of that series for use in its Monte-Carlo model run.

## 3 Results

### 3.1 D/L vs age

**Fig. 6:**
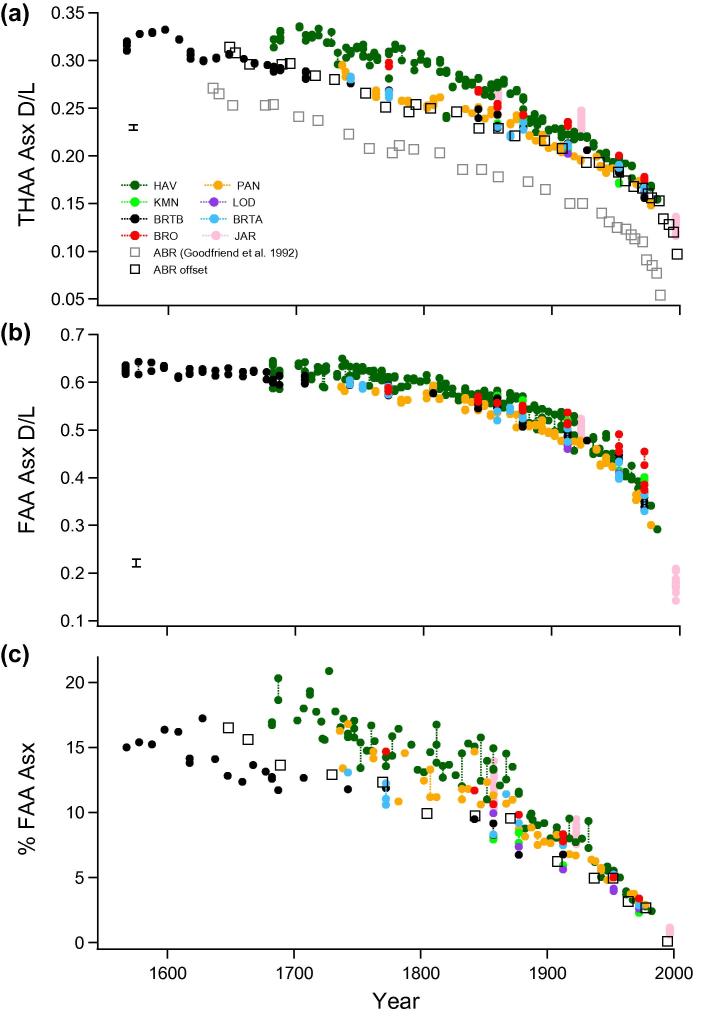
D/L of aspartic acid (Asx) in the (a) Total Hydrolysable Amino Acid (THAA) and (b) Free (FAA) fractions for all core samples vs year determined from independent dating. (c) Percentage of Asx in the FAA for coral cores vs year. The data from Abraham Reef (Goodfriend et al., 1992) is also shown for comparison; the grey squares indicate the original values, the black squares show the data corrected for time since analysis and hydrolysis protocol (13 years; Section 2.5). Data points are averages of duplicate analyses, lines between replicate samples. Error bars represent 2 stdev of mean for replicate samples.

**Fig. 7:**
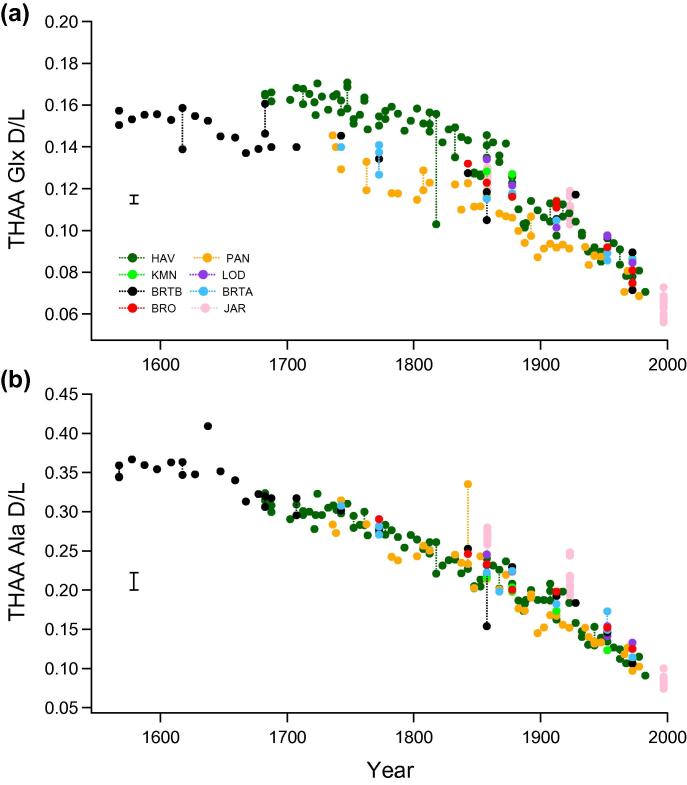
D/L of glutamic acid (Glx) and alanine (Ala) in the THAA fraction for all core samples vs year determined by independent dating. Data points are averages of duplicate analyses, lines between replicate samples. All errors bars are 2 stdev.

The ratio of d to l amino acids in the coral skeletons displayed a strong age-dependent pattern in all the *Porites* colonies (Figs. 6 and 7, EA-1 and 2). The extent of amino acid racemization was much higher in the intra-crystalline Free fraction (e.g. Asx FAA D/L Fig. 6b, also Glx, alanine (Ala), serine (Ser) FAA D/L plotted in Fig. EA-1), than in the Total Hydrolysable fraction (e.g. Asx THAA D/L Fig. 6a, Glx and Ala THAA D/L Fig. 7). This is expected, and presumably due to formation of FAA via peptide bond hydrolysis of the racemized terminal amino acids, rather than increased rates of racemization as FAA (e.g. Mitterer and Kriausakul, 1984). The initial extent of racemization between the colonies was similar and AAR values within individual colonies followed stratigraphically consistent trajectories. The extent of racemization through time was also very similar between colonies and sites, but there were slight differences in THAA Asx D/L and Glx D/L that became progressively more pronounced with ageing. There was an apparent clustering with 3 cores (HAV, BRO and JAR) following a trajectory of slightly higher levels of racemization than the remaining 5 cores and the record from Abraham Reef (Goodfriend et al., 1992). The significance of any trajectory differences were tested using the age-depth uncertainty envelopes developed from the Monte Carlo statistical modelling.

### 3.2 % Free vs age

The generation of FAA through protein hydrolysis was observed to increase steadily over time (Figs. 6c, EA-2). This result demonstrates that there has been no loss of the products of protein decomposition from the intra-crystalline AA pool, and so conforms with expectations of a closed system and its successful isolation with the bleach pre-treatment. Again, minor differences were observed in the rate of FAA generation between individual cores with those from midshelf and offshore sites (ABR, two BRT, LOD; Table 1) generating FAA at a consistently lower level than the majority of inshore GBR and equatorial (JAR) colonies (Fig. 6c). In the mid-late 19th C. a marked step in% FAA values between stratigraphically adjacent samples was also apparent for many of the individual core records, and was mirrored in THAA Asx D/L and Glx D/L trajectories (Fig. 7).

### 3.3 % Free vs D/L

**Fig. 8:**
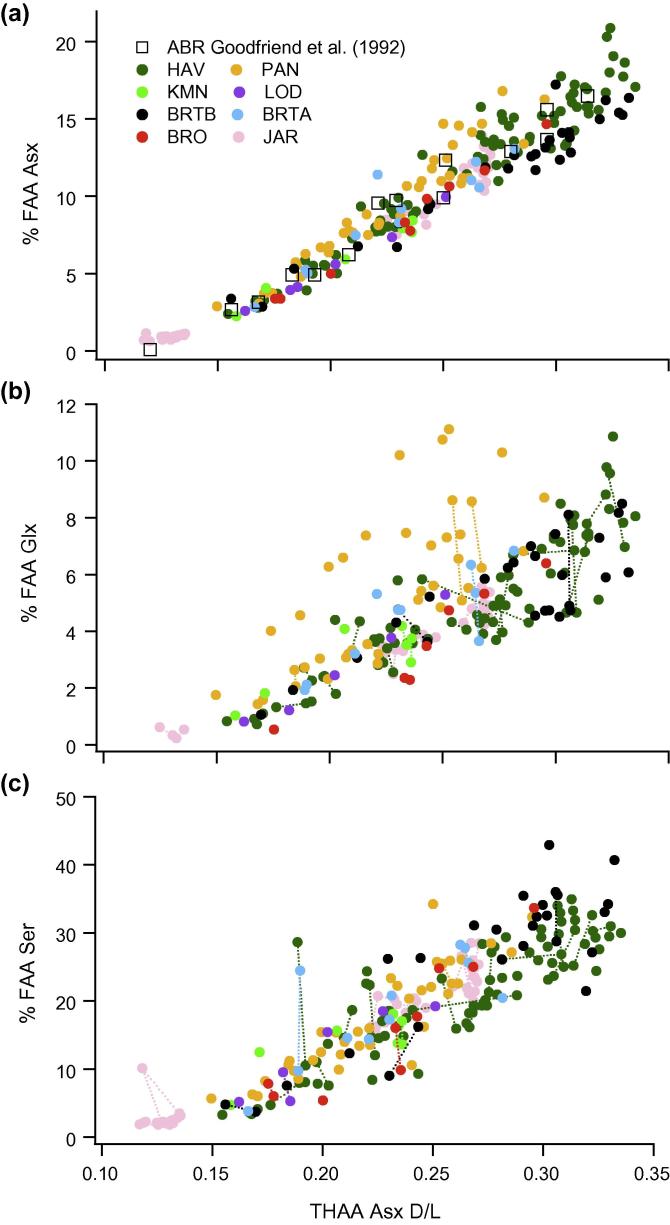
D/L Asx for the Total Hydrolysable amino acid (THAA) fraction vs % Free Asx, % Free Glx and % Free Ser. The data from Abraham Reef (Goodfriend et al., 1992) is also shown (Black squares; with D/L correction of 0.043; Section 2.5). Lines are plotted between replicated samples.

As protein degrades, progressive hydrolysis of the peptide bonds increases the number of terminal amino acids (Kriausakul and Mitterer, 1980; Mitterer and Kriausakul, 1984). Most amino acids only racemise when in a terminal position, and so AAR and protein hydrolysis are linked. Asx is an exception and can racemise within the peptide chain (Geiger and Clarke, 1987). In addition, peptide bonds containing Asx are relatively easy to hydrolyse, thereby readily generating FAAs (Schultz, 1967; Marcus, 1985). *Porites* coral intra-crystalline proteins contained a high concentration of Asx (∼50%) that ultimately leads to relatively rapid generation of FAA. Note that the anomalous % FAA values, which also poorly replicate with full repeat analysis of the sample (Fig. 8), are associated with extreme low THAA concentrations.

## 4 Discussion

The results from Goodfriend et al. (1992) showed considerable promise for the application of amino acid geochronology to dating coral material from the last few centuries. In this study we take this foundation work further by (1) incorporating recent method improvements, isolating the intra-crystalline skeletal protein and measuring by RP-HPLC, (2) assessing the variability in AAR between coral colonies, and (3) applying new Monte Carlo statistical modelling treatment of the results in order to quantify uncertainty of AAR age determinations.

### 4.1 Methodological advances

AAR closed system behaviour has been demonstrated in the intra-crystalline fraction of proteins in eggshell (Brooks et al., 1990) and mollusc shells (Penkman et al., 2008; Demarchi, 2009). The removal of extra-crystalline contaminants by 48 h exposure to bleach significantly reduced variability in both AAR and composition between sub-samples of coral (Fig. 2). Our results demonstrate that the extent of hydrolysis and racemization in the coral intra-crystalline fraction also follows ideal closed system behaviour. As a result it was possible to accurately measure Asx D/L of the intra-crystalline Free fraction, and demonstrate for the first time the sensitivity of this measurement as a dating tool in coral (Fig. 6b; Section 4.3). The analysis of multiple amino acids further extends the potential dating application of AAR in coral. The rapidly racemizing aspartic acid (Asx) enables a high degree of resolution at short timescales (Fig. 6), particularly in the FAA fraction, with slower rates for other amino acids (e.g. Glx, Ala; Fig. 7) indicating that chronologies much longer than 500 years can be developed (as illustrated in Section 4.3).

### 4.2 AAR variability between colonies

Our study is the first to assess the natural variability in intra-crystalline AAR between colonies and hence the level of dating resolution possible in coral for the last 5 centuries. Variability observed in our late-Holocene material is small in comparison with Wehmiller et al. (1976)’s analysis of multiple Pleistocene coral specimens. In this previous study, Wehmiller found poor reproducibility with less than half the samples (16 out of 38) demonstrating concordance with known age. Despite the recent methodological advances, including the isolation and analysis of a more reproducible intra-crystalline protein, it is evident that the patterns of degradation from coral colonies of the same genus within the same region are not always consistent. As a result our analysis of multiple coral colonies does demonstrate some limitations for the geochronological application of AAR in corals. Although amino acid racemization *within* a colony follows a stratigraphic-aging pattern, there are apparent differences in rates *between* the colonies (Figs. 6 and 7). The AAR trajectories do, however, appear to group into systematic patterns.

#### 4.2.1 Thermal conditions and geographical location

Even in an optimal set of samples, with closed system behaviour and identical (internal) chemical environments for degradation, aminostratigraphic correlation between sites relies on the samples sharing an equivalent temperature history (e.g. Wehmiller and Miller, 2000). The temperature effect should be most apparent between cores from the three regions (Table 1); Abraham Reef in the Southern GBR (22.10°S), ∼200 miles south of the 7 central GBR colonies (18–19°S), and the equatorial Pacific Jarvis Island site (0.37°S). Abraham Reef experienced annual mean sea surface temperatures ∼1 to 1.5 °C cooler than the central GBR colonies analysed in this study, although both share a similar seasonal range of ∼5–6 °C (Table 1; Reynolds et al., 2002). In contrast, sea surface temperatures at Jarvis Island are 1–2 °C warmer than the GBR sites with almost no seasonality (1 °C). Despite this temperature difference, the extent of racemization is very similar between all core locations (Figs. 6 and 7).

The Jarvis Island (JAR) coral shows much greater variability than that observed in any of the other coral samples, however, this reflects sampling resolution (also see EA Fig. 3). The JAR material was analysed at approximately monthly resolution, whereas all the GBR records are from homogenised bulk samples of 1–2 years growth for the Abraham Reef core (ABR), and 5-year increments for the central GBR cores. It is possible that the JAR data are capturing sub-annual variations that are smoothed out in the bulk samples from the GBR. Exposure to a seasonal cycle of 1 °C for the JAR core is unlikely to cause the extent of variability observed, considering an annual average of +1–2 °C SST does not create an equivalent offset from the other sites. Instead, sub-annual variations may be dominated by composition changes in skeletal protein over this timescale, rather than temperature-induced degradation. A further seasonality-related temperature effect is possible across the GBR continental shelf, caused by the larger SST range observed in the near- and inshore relative to mid- and offshore reef sites, although the annual SST average is the same. However assuming an effective activation energy of −121 kJ mol^−1^ the difference between the sites is only 0.15 °C, insufficient to account for the variance observed.

The extent of Asx racemization in ABR (Goodfriend et al., 1992) is consistent with a cluster of cores in our study (Figs. 6 and 7) when the date of sample analysis and the hydrolysis protocols are taken into account (calculated offset applied as prescribed in Section 2.5). When the calculated offset due to the increase in Asx D/L for the hydrolysis protocol is applied to the ABR% FAA data, the generation of Free Asx also conforms with that observed in our study (Figs. 7 and 8). As with the warmer JAR site results, there is no apparent response to suggest that the cooler temperature history at ABR had a significant impact on the extent of racemization.

#### 4.2.2 Significance in clustering of AAR trajectories

**Fig. 9:**
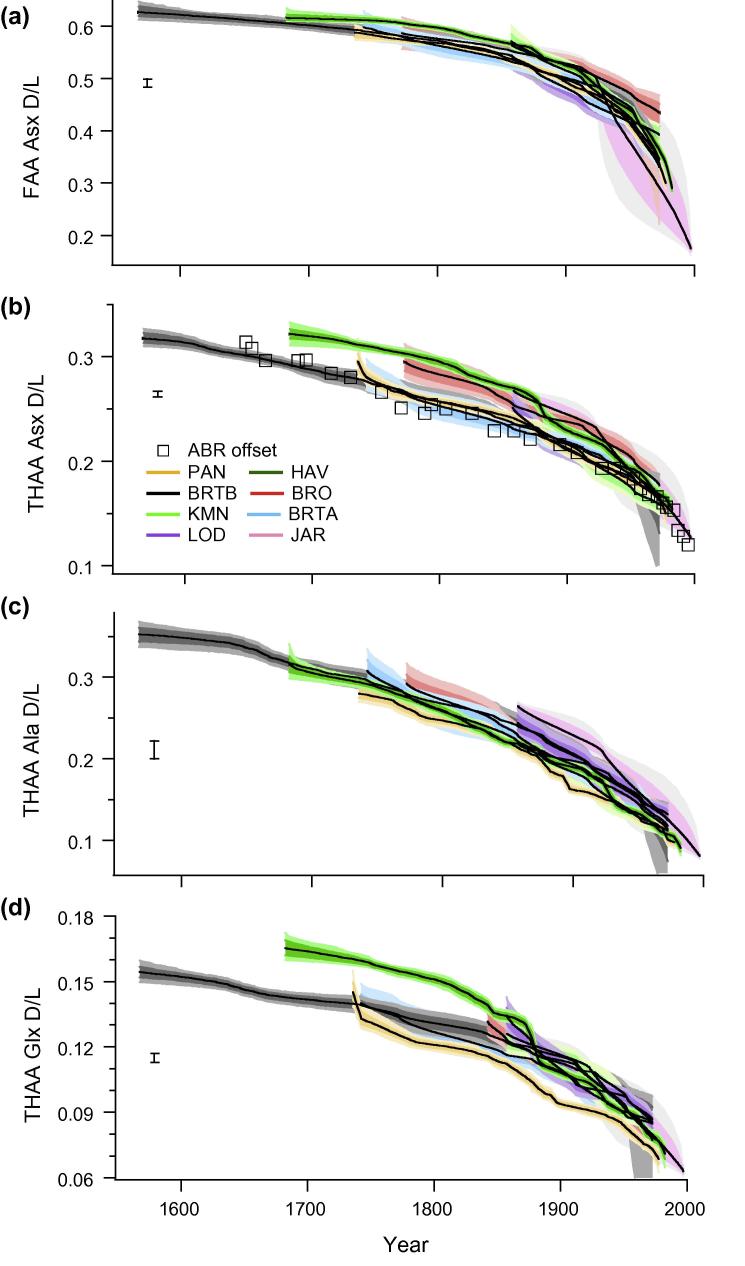
Age-depth uncertainty envelopes generated by Bayesian-Monte Carlo modelling of the AAR age estimates (50% median curve in black for each core, and 1 and 2σ confidence bands). D/L Asx for the (a) Free (FAA) and (b) Total Hydrolysable amino acid (THAA) fractions, (c) D/L of alanine (Ala) and (d) glutamic acid (Glx) THAA fractions. All errors bars are 2 stdev. The corrected (Section 2.5) data from Abraham Reef (ABR; Goodfriend et al., 1992) is also shown (Black squares).

Age-depth uncertainty envelopes were generated for each core from least square Monte Carlo modelling of the AAR age estimates (Section 2.6; Fig. 9). This enables the apparent clustering of trajectories between coral colonies to be objectively explored and statistically demonstrated within 1 and 2σ confidence bands. For FAA Asx D/L (Fig. 9a) all corals follow the same racemization trend within error, with the exception of the two most heavily sampled cores (PAN; yellow and HAV; dark green) where the latter shows a significantly faster rate of degradation. On the other hand, due to greater precision on the THAA Asx D/L values, the uncertainty envelopes do not overlap all cores and instead reveal statistically significant separation between individual trajectories (Fig. 9b). Two unique trajectories can be seen with a lower cluster of cores (BRTA, BRTB, PAN, LOD and KMN) conforming strongly to the ABR results. This grouping is not collective around any obvious environmental factor and contains coral collected from nearshore to offshore, and central to southern GBR sites. The only characteristic shared by the 3 remaining cores that we could identify is that all are from island fringing reefs, although there is no clear reasoning why such an environment should be a significant factor in THAA Asx D/L. Jarvis Island is truly oceanic, being atop a seamount well removed from any source of terrestrial runoff or other influence (core JAR). Both Brook and Havannah Islands are high continental islands within the inshore GBR lagoon (cores BRO and HAV).

The most consistent inshore-offshore gradients in AAR are seen in %FAA and THAA Ala D/L (Figs. 6c and 7b). The THAA Ala D/L curves spread out along an offshore environmental gradient (Fig. 9c), with the nearshore and continental fringing reef cores (PAN, HAV, KMN) consistently displaying lower initial extent of Ala racemization and the most oceanic site (JAR) the highest D/L values. Entrapped protein may, therefore, be a useful source of additional environmental information, although further study is needed to determine the cause of this response.

In contrast, the THAA Glx D/L records demonstrate a further pattern, with the two closest core sites (PAN; yellow and HAV; green) racemizing at significantly different rates for this amino acid, and PAN at significantly slower rate than all other cores. Retroflections are particularly marked in these same two cores across all AAR results, but most obviously in the acidic AAs as demonstrated in the THAA Glx D/L plot (Fig. 9d). The rate change is slightly offset in the time domain, around 1900 in PAN (Fig. 9; yellow curves), and 1870–80 in HAV (dark green curves). Significant changes in environmental conditions are recorded in the late 19th C in the GBR (e.g. Hendy et al., 2002; Lewis et al., 2007), but further investigation is needed to isolate what factor could be influencing AAR rates. AAR from duplicate BRT cores (central GBR) are statistically inseparable despite collection 4 years apart and, therefore, potential differences caused by subsequent storage environments and treatment.

Since differences are found between cores of massive *Porites* sp. coral from different colonies, it is unsurprising that significant differences were observed previously in the rates and patterns of racemization from different coral genera (Wehmiller et al., 1976; Nyberg et al., 2001). The cause of the differences in protein breakdown within and between genera is an interesting question for further investigation; possible reasons include differences in protein composition and seasonality of the composition incorporated into the skeleton, differences in the physico-chemical micro-environment within the intra-crystalline pores, to physiological differences between coral colonies related to species, algal symbionts or gender. The utility of this dating method has, however, been tested at the level of taxonomic and physiological detail possible in applications using fossil material.

### 4.3 Application of AAR and Monte Carlo statistical modelling

**Fig. 10:**
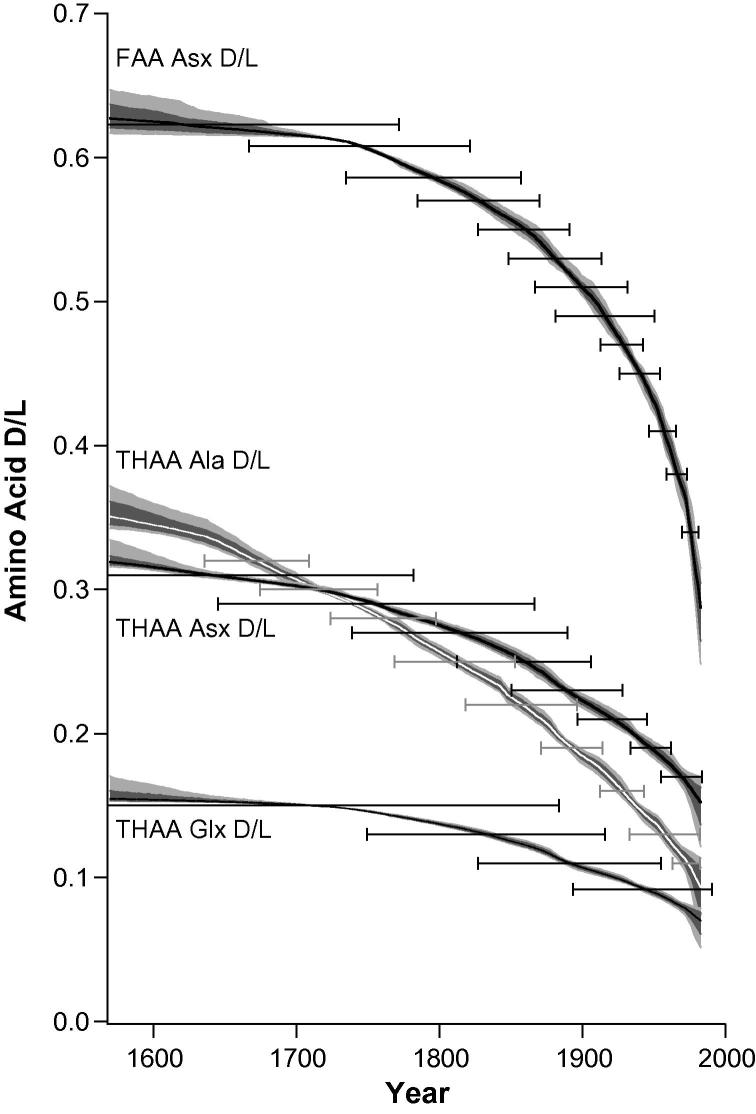
Age-depth uncertainty envelopes generated by Bayesian-Monte Carlo modelling of the AAR age estimates from all GBR samples combined (50% median curve in black, and 1 and 2σ confidence bands) for Free (FAA) D/L Asx and Total Hydrolysable amino acid (THAA) fractions of D/L Asx, alanine (Ala) and glutamic acid (Glx). Error bars are 2σ (in years) of the population of independently ascribed ages found with a selected range of measured D/L values, and represent the estimated dating error that would be applied to analyses of unknown age.

The uncertainty associated with each AAR age determination is formally quantified by the least square Monte Carlo statistical modelling (Section 2.6; Fig. 9). The Free Asx D/L age estimations are clearly the most promising universal AAR dating tool (Fig. 9a), especially for previously un-analysed colonies, because of the insignificant differences observed between trajectories from different colonies irrespective of temperature histories and geographical locations. Free Asx D/L could be used for geochronology in corals with the average 2σ precision for material laid down within the last 150 years of <10 years obtained for individual colonies (calculated for HAV). When a single curve is modelled for all the Free Asx D/L data combined an unknown sample from the last 150 years could be dated with the precision of ±24 years (2σ; Fig. 10).

This study has demonstrated that internally consistent patterns of protein breakdown occur within *Porites* coral colonies over the timescale of the last 500 years, but differences are observed in the THAA degradation patterns between colonies (Fig. 9b–d) and this decreases the widespread utility of this fraction for geochronology. With the isolation of the intra-crystalline fraction and the increased sensitivity of new analytical techniques 2σ age error estimates of ±12, 12 and 14 years for THAA Asx D/L, Ala DL and Glx D/L respectively can be obtained in 20th C material (calculated using data from HAV and taking the average of modelled 2σ estimates; Fig. 9). If the pattern of protein breakdown for a colony is not known, then the precision of this technique as a method for dating coral samples of unknown age will be decreased because the uncertainty incorporates the AAR spread observed across the multiple colonies (Fig. 10). In this case the 2σ uncertainty using THAA Asx D/L is on the order of ±19 years over the 20th C, increasing to ±118 years in the 17th C; for THAA Ala D/L these values are ±29 years over the 20th C, increasing to ±40 years during the 17th C. This result highlights the value of measuring a suite of AAs.

The levels of precision obtained using Free Asx D/L measurements show the best dating potential over the most recent 150 years of coral growth, and may provide a useful check on other dating techniques of U/Th and annual band-counting. Zhao et al. (2009) recently reviewed the accuracy of ^230^Th ages for young corals in comparison with chronologies independently derived from band counting or δ^18^O-based wiggle-matching (e.g. Cobb et al., 2003; Shen et al., 2008). Precisions of 1–10 years (2σ) have been achieved in pristine coral samples with low initial ^230^Th dating from the last 500 years (Shen et al., 2008; Zhao et al., 2009). However, the presence of anomalous ^230^Th/^232^Th ratios can induce greater variance (e.g. up to 23 years; Shen et al., 2008). Annual band counting has also been demonstrated to have errors on the order of a decade at this time scale (Hendy et al., 2003), and particular issues arise where there are breaks in the record, resulting in a floating chronology. In addition, annual markers may not be present in all corals; for example, the luminescent bands in near-shore massive corals are much weaker offshore making cross-dating more difficult (Hendy et al., 2003), and are missing from slow-growing species (Burgess et al., 2009). The precision of Free Asx D/L geochronology is, therefore, within that obtained for difficult-to-date samples using other techniques and so would be a useful complementary and independent estimate of age. AAR could make the most valuable contribution, however, in studies that are attempting to extend coral-based climate reconstructions using recently dead and fossil coral colonies cross-dated with samples extracted from living colonies (e.g. McGregor et al., 2011). In the field, it is often very difficult to assess likely age of such material which can contain mixed populations of recent to centuries and millennia since death. It would, therefore, be extremely useful to have such an efficient, cost-effective and reliable analytical method to screen potential material and optimise sample selection for further detailed dating and climate-reconstruction labwork. AAR can also provide rapid and cheap chronologies for coral cores, as studies can be undertaken on small samples (∼3 mg), enabling high-resolution records.

## 5 Conclusions

In conclusion, the extent of hydrolysis and racemization in the intra-crystalline fraction extracted from *Porites* coral skeletons follows ideal behaviour. By isolating the intra-crystalline fraction through bleach treatment, and following strict data selection protocols, 2σ precision on age estimates as low as ±6 years can be achieved from the Free Asx D/L fraction in the most recent coral material, and ±24 years for material of up to 150 years age. This is the first study to demonstrate that patterns of protein breakdown differ over time between massive *Porites* colonies of the same or closely related species of coral. The question of why rates of decay differ, irrespective of shared temperature histories and geographical locations, raises interesting questions about what factors control the composition of the intra-crystalline protein deposited by the coral. The significant value of amino acid racemization in coral is its utility as a cheap and rapid geochronological tool for screening populations of coral samples in order to identify those for further investigation.

## Figures and Tables

**Table 1:** Core sample sites; 8 from this study and Abraham Reef (Goodfriend et al., 1992). The cores from the Great Barrier Reef were collected by the Australian Institute of Marine Sciences. Mean annual SST from the NOAA NCEP EMC CMB GLOBAL Ov2 climatology for 1961–1990 (Reynolds et al., 2002).

Coral locality	Latitude (°S)	Longitude (°E)	Distance from continental land mass (km)	Core code	Date collected	Earliest date analysed in core (age in years)	Mean annual SST (°C)
*Central Equatorial Pacific*							
Jarvis Is.	0.37	200.02	>1000	JAR	Sept 1997	1858 (141)	27.1

*Central Great Barrier Reef*							
Kurrimine Rf.	17.78	146.13	0 (inshore)	KMN	June 1988	1850 (138)	26.4
Brook Is.	18.09	146.17	29 (inshore)	BRO	May 1987	1755 (232)	26.2
Britomart Rf.	18.14	146.44	36 (midshelf)	BRT(A) BRT(B)	July 1984 May 1987	1740 (244) 1565 (422)	26.2
Lodestone Rf.	18.42	147.06	70 (midshelf)	LOD	July 1984	1850 (134)	26.1
Pandora Rf.	18.82	146.43	16 (inshore)	PAN	Mar 1984	1735 (249)	26.2
Havannah Is.	18.85	146.55	24 (inshore)	HAV	June 1988	1680 (308)	26.2

*Southern Great Barrier Reef*							
Abraham Reef	22.10	152.50	195 (offshore)	ABR	Dec 1985	1632 (353)	24.9

## References

[b0005] Abram N.J., Gagan M.K., Cole J.E., Hantoro W.S., Mudelsee M. (2008). Recent intensification of tropical climate variability in the Indian Ocean. Nat. Geosci..

[b0010] Abram N.J., McGregor H.V., Gagan M.K., Hantoro W.S., Suwargadi B.W. (2009). Oscillations in the southern extent of the Indo-Pacific Warm Pool during the mid-Holocene. Quat. Sci. Rev..

[b0015] Bada J.L. (1971). Kinetics of non-biological decomposition and racemization of amino acids in natural waters. Amer. Chem. Soc. Adv. Chem. Ser..

[b0020] Brooks A.S., Hare P.E., Kokis J.E., Miller G.H., Ernst R.D., Wendorf F. (1990). Dating Pleistocene archaelogical sites by protein diagenesis in ostrich eggshell. Science.

[b0025] Burgess S.N., McCulloch M.T., Mortimer G.E., Ward T.M. (2009). Structure and growth rates of the high-latitude coral: *Plesiastrea versipora*. Coral Reefs.

[b0030] Cobb K.M., Charles C.D., Cheng H., Edwards R.L. (2003). El Niño/Southern Oscillation and tropic Pacific climate during the last millennium. Nature.

[b0035] Collins M.J., Riley M.S., Goodfriend G.A., Collins M.J., Fogel M.L., Macko S.A., Wehmiller J.F. (2000). Amino acid racemization in biominerals, the impact of protein degradation and loss. Perspectives in Amino Acid and Protein Geochemistry.

[b0040] Collins M.J., Waite E.R., van Duin A.C.T. (1999). Predicting protein decomposition: the case of aspartic-acid racemization kinetics. Philos. Trans. R. Soc. Lond. Ser. B-Biol. Sci..

[b0050] Csapo J., CsapoKiss Z., Wagner L., Talos T., Martin T.G., Folestad S., Tivesten A., Nemethy S. (1997). Hydrolysis of proteins performed at high temperatures and for short times with reduced racemization, in order to determine the enantiomers of d- and l-amino acids. Anal. Chim. Acta.

[b0055] Demarchi B. (2009). Geochronology of Coastal Prehistoric Environments: A New Closed System Approach Using Amino Acid Racemization.

[b0060] Demarchi B., Rogers K., Fa D. A., Finlayson C. J., Milner N. and Penkman K. E. H. (in press) Intra-crystalline protein diagenesis (IcPD) in Patella vulgata. Part 1: Isolation and testing of the closed system. Quat. Geochronol. http://dx.doi.org/10.1016/j.quageo.2012.03.016. 10.1016/j.quageo.2012.03.016 PMC3743299 23956807

[b0065] Drysdale R.N., Zanchetta G., Hellstrom J.C., Fallick A.E., Zhao J. (2005). Stalagmite evidence for the onset of the Last Interglacial in southern Europe at 129 +/− 1 ka. Geophys. Res. Lett..

[b0070] Drysdale R.N., Zanchetta G., Hellstrom J.C., Fallick A.E., Zhao J.-X., Isola I., Bruschi G. (2004). Palaeoclimatic implications of the growth history and stable isotope (δ^18^O and δ^13^C) geochemistry of a Middle to Late Pleistocene stalagmite from central-western Italy. Earth Planet. Sci. Lett..

[b0075] Gale S.J. (2009). Dating the recent past. Quat. Geochronol..

[b0305] Geiger, T. and Clarke, S. (1987) Deamidation, isomerization and racemization at asparaginyl and aspartyl residues in peptides. Succinimide-linked reactions that contribute to protein degradation. . 3805008

[b0085] Goodfriend G.A., Collins M.J., Fogel M.L., Macko S.A., Wehmiller J.F. (2000). Perspectives in Amino Acid and Protein Geochemistry.

[b0090] Goodfriend G.A., Hare P.E., Druffel E.R.M. (1992). Aspartic-acid racemization and protein diagenesis in corals over the last 350 years. Geochim. Cosmochim. Acta.

[b0095] Hendy, E. J. (2003) Coral reconstructions of decadal-to-centennial climate variability in the Great Barrier Reef since 1565AD. Ph. D. thesis, The Australian National University.

[b0100] Hendy E.J., Gagan M.K., Alibert C.A., McCulloch M.T., Lough J.M., Isdale P.J. (2002). Abrupt decrease in tropical Pacific sea surface salinity at end of Little Ice Age. Science.

[b0105] Hendy E.J., Gagan M.K., Lough J.M. (2003). Chronological control of coral records using luminescent lines and evidence for non-stationary ENSO teleconnections in northeast Australia. The Holocene.

[b0110] Hill R.L. (1965). Hydrolysis of proteins. Adv. Protein Chem..

[b0115] Husseini S.I. (1973). Temporal and diagenetic modifications of the amino acid composition of Pleistocene coral skeletons.

[b0120] Ingalls A.E., Lee C., Druffel E.R.M. (2003). Preservation of organic matter in mound-forming coral skeletons. Geochim. Cosmochim. Acta.

[b0125] Isdale P. (1984). Fluorescent bands in massive corals record centuries of coastal rainfall. Nature.

[b0130] Kaufman D.S. (2003). Dating deep-lake sediments by using amino acid racemization in fossil ostracodes. Geology.

[b0135] Kaufman D.S. (2006). Temperature sensitivity of aspartic and glutamic acid racemization in the foraminifera Pulleniatina. Quat. Geochronol..

[b0140] Kaufman D.S., Manley W.F. (1998). A new procedure for determining DL amino acid ratios in fossils using reverse phase liquid chromatography. Quat. Sci. Rev..

[b0145] Knutson R.A., Buddemeier R.W., Smith S.V. (1972). Coral chronometers: seasonal growth bands in reef corals. Science.

[b0150] Kosnik M.A., Kaufman D.S. (2008). Identifying outliers and assessing the accuracy of amino acid racernization measurements for geochronology: II. Data screening. Quat. Geochronol..

[b0155] Kosnik M.A., Kaufman D.S., Hua Q. (2008). Identifying outliers and assessing the accuracy of amino acid racemization measurements for geochronology: I. Age calibration curves. Quat. Geochronol..

[b0160] Kriausakul N., Mitterer R.M., Hare P.E., Hoering T.C., King K.J. (1980). Some factors affecting the epimerisation of isoleucine in peptides and proteins. Biogeochemistry of amino acids.

[b0165] Laabs B.J.C., Kaufman D.S. (2003). Quaternary highstands in Bear Lake Valley, Utah and Idaho. Geol. Soc. Am. Bull..

[b0170] Lewis S.E., Shields G.A., Kamber B.S., Lough J.M. (2007). A multi-trace element coral record of land-use changes in the Burdekin River catchment, NE Australia. Paleogeogr. Paleoclimatol. Paleoecol..

[b0175] Lough J.M., Barnes D.J. (1992). Comparisons of skeletal density variations in *Porites* from the Central Great-Barrier-Reef. J. Exp. Mar. Biol. Ecol..

[b0180] Lough J.M., Barnes D.J. (2000). Environmental controls on growth of the massive coral *Porites*. J. Exp. Mar. Biol. Ecol..

[b0185] Lough J.M. (2011). Great Barrier Reef coral luminescence reveals rainfall variability over northeastern Australia since the 17th century. Paleoceanography.

[b0190] Marcus F. (1985). Preferetial cleavage at Aspartyl-prolyl peptide-bonds in dilute acid. Int. J. Pept. Protein Res..

[b0195] McGregor H.V., Hellstrom J., Fink D., Hua Q., Woodroffe C.D. (2011). Rapid U-series dating of young fossil corals by laser ablation MC-ICPMS. Quat. Geochronol..

[b0200] Miller G.H., Brigham-Grette J. (1989). Amino acid geochronology: resolution and precision in carbonate fossils. Quatern. Int..

[b0205] Mitterer R.M., Kriausakul N. (1984). Comparison of rates and degrees of isoleucine epimerization in dipeptides and tripeptides. Org. Geochem..

[b0210] Nyberg J., Csapo J., Malmgren B.A., Winter A. (2001). Changes in the d- and l-content of aspartic acid, glutamic acid, and alanine in a scleractinian coral over the last 300 years. Org. Geochem..

[b0215] Penkman, K. E. H. (2005) Amino acid geochronology: a closed system approach to test and refine the UK model. Unpublished PhD thesis, University of Newcastle.

[b0220] Penkman K.E.H., Kaufman D.S., Maddy D., Collins M.J. (2008). Closed-system behaviour of the intra-crystalline fraction of amino acids in mollusc shells. Quat. Geochronol..

[b0225] Preece R.C., Penkman K.E.H. (2005). New faunal analyses and amino acid dating of the Lower Palaeolithic site at East Farm, Barnham, Suffolk. Proc. Geol. Assoc..

[b0230] Radkiewicz J.L., Zipse H., Clarke S., Houk K.N. (1996). Accelerated racemization of aspartic acid and asparagine residues via succinimide intermediates: an ab initio theoretical exploration of mechanism. J. Am. Chem. Soc..

[b0235] Reynolds R.W., Rayner N.A., Smith T.M., Stokes D.C., Wang W. (2002). An improved in situ and satellite SST analysis for climate. J. Climate.

[b0240] Schultz J. (1967). Cleavage at aspartic acid. Methods Enzymol..

[b0245] Shen C.-C., Li K.-S., Sieh K., Natawidjaja D., Cheng H., Wang X., Edwards R.L., Lam D.D., Hsieh Y.-T., Fan T.-Y., Meltzner A.J., Taylor F.W., Quinn T.M., Chiang H.-W., Kilbourne K.H. (2008). Variation of initial ^230^Th/^232^Th and limits of high precision U–Th dating of shallow-water corals. Geochim. Cosmochim. Acta.

[b0250] Steinberg S., Bada J.L. (1981). Diketopiperazine formation during investigations of amino-acid racemization in dipeptides. Science.

[b0255] Sykes G.A., Collins M.J., Walton D.I. (1995). The significance of a geochemically isolated intracrystalline organic fraction within biominerals. Org. Geochem..

[b0265] Towe K.M., Hare P.E., Hoering T.C., King K.J. (1980). Preserved organic ultrastructure: an unreliable indicator for Paleozoic amino acid biogeochemistry. Biogeochemistry of Amino Acids.

[b0270] Towe K.M., Thompson G.R. (1972). The structure of some bivalve shell carbonates prepared by ion beam thinning. Calcif. Tissue Res..

[b0275] Tudhope A.W., Chilcott C.P., McCulloch M., Cook E.R., Chappell J., Ellam R.M., Lea D.W., Lough J.M., Shimmield G.B. (2001). Variability in the El Niño-Southern Oscillation through a glacial-interglacial cycle. Science.

[b0280] Tudhope A.W., Shimmield G.B., Chilcott C.P., Jebb M., Fallick A.E., Dalgleish A.N. (1995). Recent changes in climate in the far western equatorial Pacific and their relationship to the Southern Oscillation; oxygen isotope records from massive corals, Papua New Guinea. Earth Planet. Sci. Lett..

[b0285] Wehmiller J.F., Hare P.E., Kujala G.A. (1976). Amino acids in fossil corals: racemization (epimerization) reactions and their implications for diagenetic models and geochronological studies. Geochim. Cosmochim. Acta.

[b0290] Wehmiller J.F., Miller G.H., Noller J.S., Sowers J.M., Lettis W.R. (2000). Aminostratigraphic dating methods in Quaternary geology. Quaternary Geochronology: Methods and Applications.

[b0295] Yu K.-F., Zhao J.-X., Shi Q., Chen T.-G., Wang P.-X., Collerson K.D., Liu T.-S. (2006). U-series dating of dead *Porites* corals in the South China sea: evidence for episodic coral mortality over the past two centuries. Quat. Geochronol..

[b0300] Zhao J.X., Yu K.F., Feng Y.X. (2009). High-precision U-238-U-234-Th-230 disequilibrium dating of the recent past: a review. Quat. Geochronol..

