# Supplementary material for: Assessing amino acid racemization variability in coral intra-crystalline protein for geochronological applications
Source: Geochim Cosmochim Acta. 2012 Jun 1;86(9-2):338–53. doi: 10.1016/j.gca.2012.02.020 (PMC3617617; doi:10.1016/j.gca.2012.02.020)
Supplement: Supplementary Figs. 1–3 [file mmc1.doc]

**SUPPLIMENTARY INFORMATION (Electronic Annex)**

EA Figure 1. Average Glx D/L, Ala D/L and Ser D/L of the FAA fraction, and Ser D/L of the THAA fraction for coral cores vs year determined independently. Data points are averages of duplicate analyses, lines between replicate samples.

EA Figure 2. Percentage of Glx and Ser in the FAA fraction for coral cores vs year determined independently. Data points are averages of duplicate analyses, lines between replicate samples.

EA Figure 3. Jarvis Island (JAR) high resolution FAA Asx D/L, THAA Asx D/L, Glx D/L and Ala D/L. Data points are averages of duplicate analyses.

**EA Figure 1.**


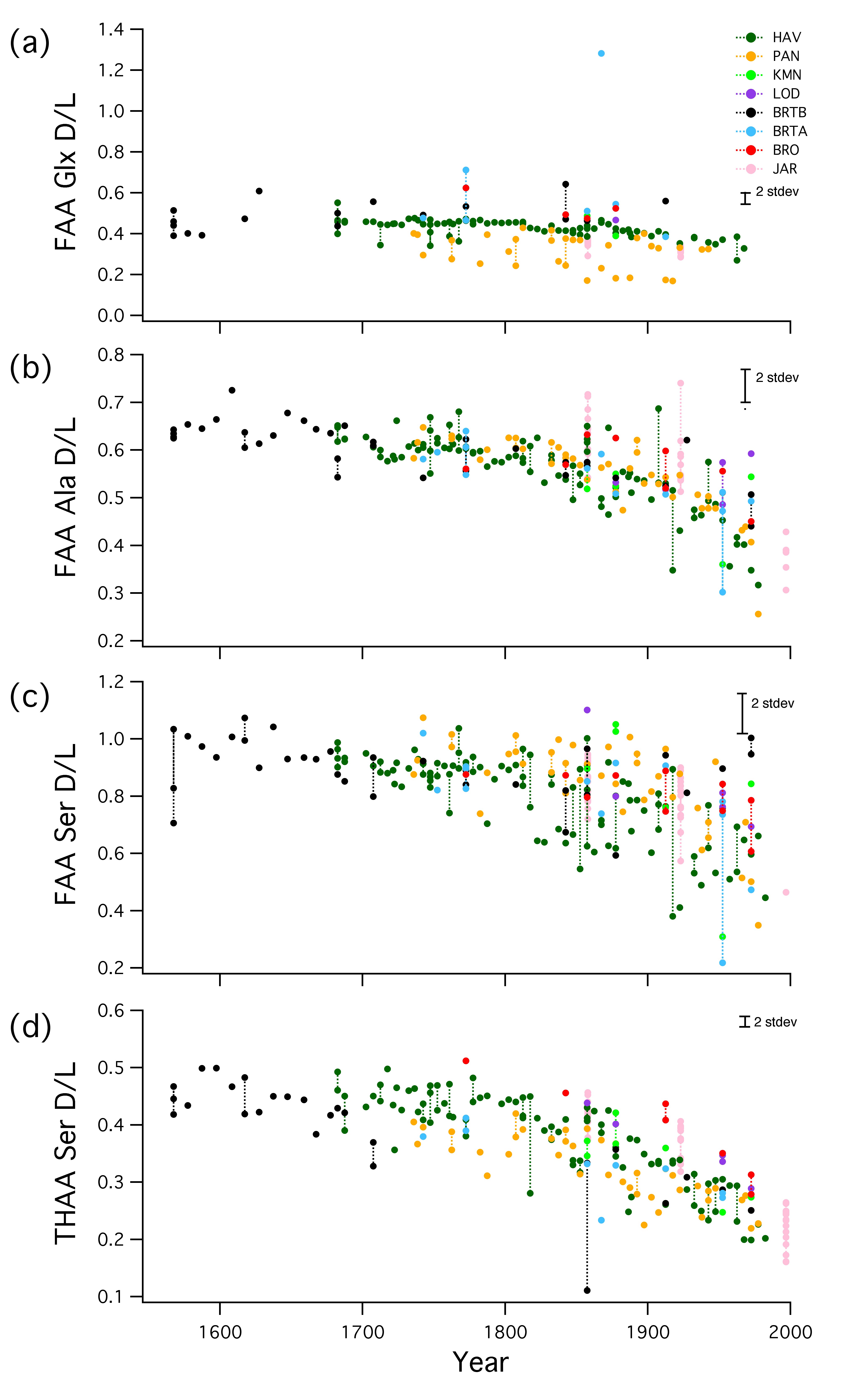


**EA Figure 2.**

######
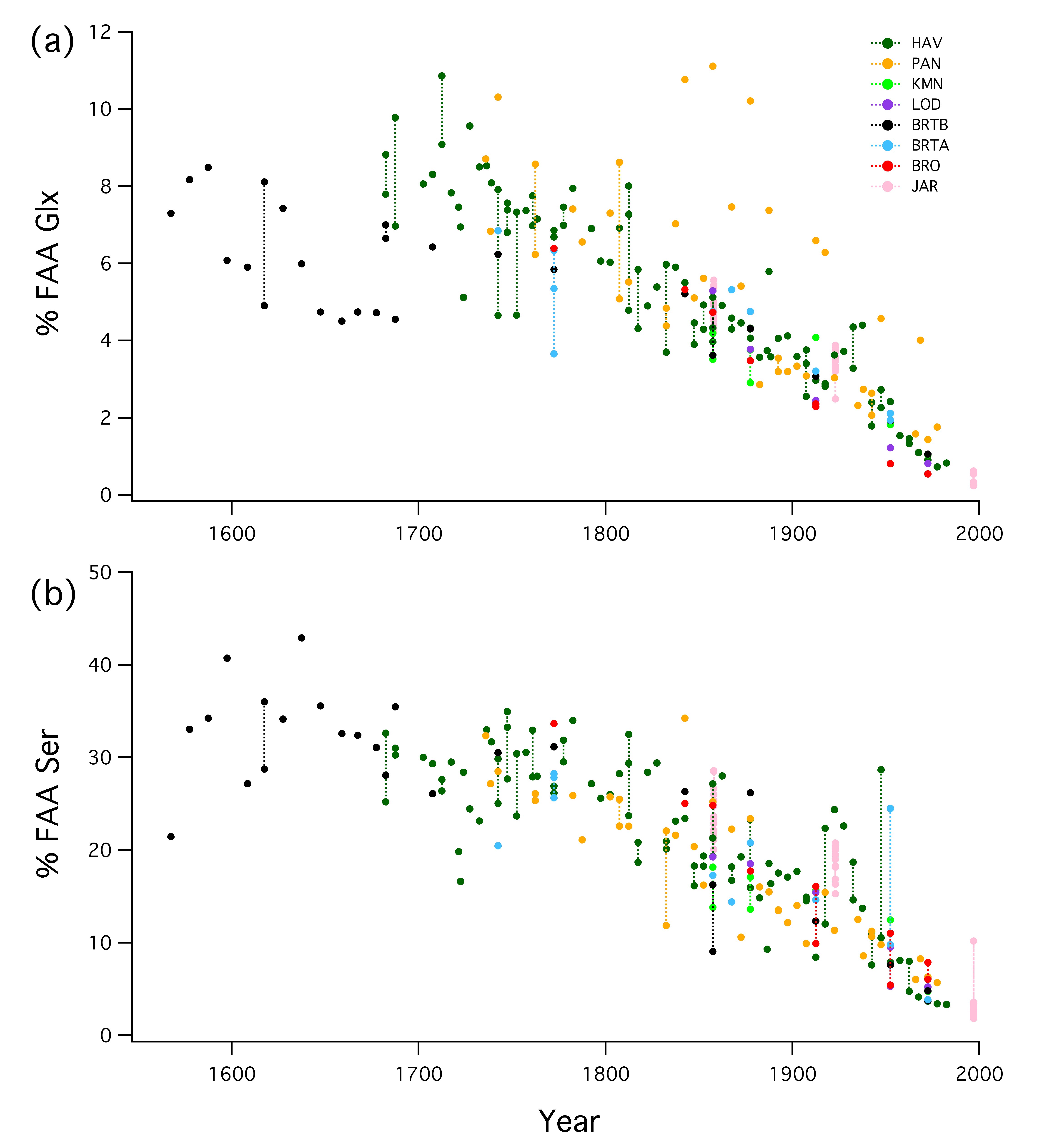


**EA Figure 3.**

**EA1.** **Further analysis of concentration data and cut-off values:**

Samples with abnormal concentrations in any of the 12 amino acids were also investigated and two identified as compromised (Fig. 4b). The two analytical replicates both showed comparably low concentrations of Glx and Asx, so plotted close to the line of best-fit and were not identified in the previous statistical tests as outliers (Fig. 4a).

The best cut-off value (2.5 stdev or 2 stdev) was also examined by looking at the concentration data of previously explored outliers. In each case the data points highlighted as >2.5 stdev away from the regression line clearly displayed abnormal THAA concentrations. However, in some of the samples with residuals between the 2-2.5stdev bound, the THAAs did not appear abnormal. The cut-off value of 2.5 stdev was therefore chosen as the most consistent criteria to exclude ‘anomalous’ data.

Figure EA 4 (a) THAA Asx concentration vs Glx concentration, showing the two data-points that were not identified as unusual using the outlier detection method detailed in section 2.4.2. (b) The same data-points highlighted in (a) show a clear drop in concentration for multiple amino acids. Only 6 of the12 amino acids have been shown for clarity. All data-points in this example are Jarvis core samples.

**EA 2. Data as Excel spreadsheet**
